# Neuropsychiatry of atrial fibrillation: dementia and beyond

**DOI:** 10.3389/fcvm.2025.1485837

**Published:** 2025-02-20

**Authors:** Mayuresh Chaudhari, Juan Rodriguez, Alejandro Velasco, Ildiko Agoston, Sudha Seshadri, Antonio L. Teixeira

**Affiliations:** ^1^ The Glenn Biggs Institute for Alzheimer’s & Neurodegenerative Diseases, The University of Texas Health Science Center at San Antonio, San Antonio, TX, United States; ^2^ Janey & Dolph Briscoe Division of Cardiology, Department of Medicine, The University of Texas Health Science Center at San Antonio, San Antonio, TX, United States

**Keywords:** atrial fibrillation, dementia, stress, anxiety, depression, autonomic regulation

## Abstract

Atrial fibrillation (AF) is the most frequent heart rhythm disorder worldwide with a prevalence of 1%–2% in general population. It is associated with increased mortality and morbidity, including increased risk of dementia. In addition to cognitive impairment, AF has been related to anxiety and mood disorders. Herein we review the literature on the association between AF and neuropsychiatric conditions, including anxiety and mood disorders. The mechanisms underlying the association between AF and dementia are complex, including stroke, chronic cerebral hypoperfusion, and systemic inflammation. There is a bidirectional interaction between AF and anxiety/mood disorders with shared mechanisms involving dysfunction of the autonomic, neuroendocrine and immune systems. Optimizing pharmacological treatment, avoiding drug interactions and implementing behavioral interventions can have a lasting impact on patients with AF undergoing rhythm/rate control therapies and/or catheter ablation.

## Introduction

Atrial fibrillation (AF) is the most frequent heart rhythm disorder worldwide with a prevalence of 1%–2% in the general population and a greater prevalence (8%–10%) in older adults (1). The number of AF in adults >55 years of age was estimated around 8.8 million in 2010 and is projected to rise to 17.9 million in 2060 (2). In addition to age, hypertension, diabetes mellitus, obesity, chronic obstructive pulmonary disease, excessive alcohol consumption, valvular heart disease, sleep apnea and obesity are known risk factors for AF (3). The lifetime risk of AF has been estimated to be 1 in 3 among American white people and 1 in 5 among African Americans (4).

AF is a chronic condition associated with increased mortality and morbidity, including a five-fold increased risk for stroke. Factors that increase risk for stroke are usually accounted for in clinical scores such as the CHA_2_DS_2_Vasc take considers age, hypertension, congestive heart failure, diabetes, vascular disease and history of prior strokes. A systematic review of prospective studies found a wide variability in stroke risk among patients with AF, ranging from 0.45%–9.28% per year (5). This variability reflects the role played by multiple factors in the pathogenesis of AF, including atrial dilatation and remodeling, subtherapeutic anticoagulation, incomplete ablation with reemergence of AF, and suboptimal drug therapy for maintenance of sinus rhythm (6). Rhythm control of AF is challenging despite various treatment options, with an estimated 30%–60% recurrence rate (6).

In addition to its thromboembolic risk, AF is a risk factor for dementia (7). Although a significant portion of this risk is attributable to cerebrovascular thromboembolic events leading to vascular cognitive impairment (2), AF has been shown to be a risk factor for cognitive impairment and dementia independent of stroke. There is also growing evidence supporting a bidirectional interaction between psychiatric disorders and AF (8).

The objective of this review is to summarize the evidence of increased risk of neuropsychiatric disorders with AF and the underlying pathophysiological mechanisms. A comprehensive literature review was performed using Scopus and PubMed databases covering the period from January 1, 2000, to May 31, 2024. The electronic search focused on peer-reviewed articles in English language, deemed to be in line with the scope of this paper. The selected articles were further narrowed down based on keyword specifics and a preview of the abstracts. The keywords included atrial fibrillation, dementia, cognitive decline, anxiety, depression, post-traumatic stress disorder (PTSD), autonomic regulation.

## Atrial fibrillation and dementia

Several studies have evaluated the association between AF and dementia. While stroke is a major driver of this association, it does not fully account for it. In the Cardiovascular Health Study, among patients who were free of AF and had no history of stroke at baseline, 10.7% developed incident AF during a mean follow-up of 7 years (4). Mean cognitive scores declined faster after incident AF compared with those with no history of AF. Thus, in the absence of a previous stroke, patients with incident AF may reach thresholds of cognitive impairment or dementia at earlier ages than patients with no history of AF. The prospective Intermountain Heart Collaborative Study also showed a significantly increased risk of dementia in patients with AF [HR 1.36 (95% CI 1.1–1.6)] (9). In the Framingham Heart Study original and offspring cohorts, after adjustment for vascular risk factors and apolipoprotein E4 (a well-known genetic risk factor for Alzheimer's disease), AF was significantly associated with longitudinal decline in executive function (10).

A systematic review by Santangeli et al. including eight prospective observational studies with more than 77,000 patients, of whom 11,700 (17%) had AF (11), showed that AF was significantly and independently associated with increased rates of incident dementia (HR: 1.4;95% CI 1.2–1.7). A larger systematic review by Kalantarian et al. evaluated the association of AF with cognitive impairment or dementia, including prospective and non-prospective data (12). Overall, AF was associated with a more than two-fold increase in the risk of developing post-stroke cognitive impairment or dementia [RR (95%) = 2.7 (1.82, 4.00)]. Importantly, there was a marked increase in cognitive decline regardless of stroke history [RR 1.40 (95% CI 1.19–1.64)] (12). Taken together, evidence indicates strong association between AF and dementia independent of stroke.

**Figure 1:**
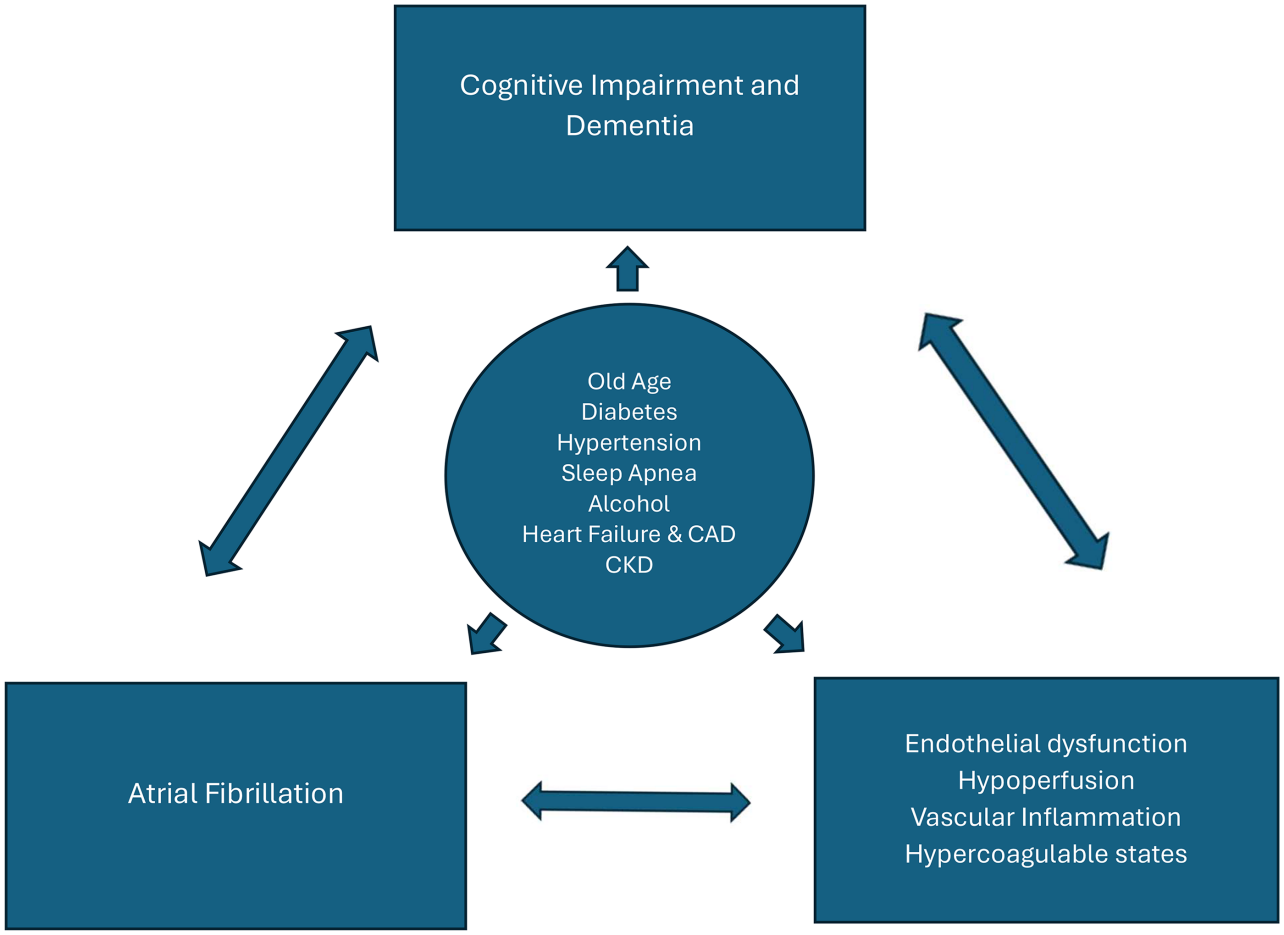
Bidirectional relationship between atrial fibrillation (AF) and cognitive impairment/dementia. Cardiovascular risk factors (circle) are associated with the development of both AF and dementia. Multiple pathophysiological mechanisms are shared by these conditions (e.g., inflammation, coagulation dysfunction) and contribute to their further progression. CAD, Coronary artery disease; CKD, chronic kidney disease.

The mechanisms underlying the link between dementia and AF independent of stroke remain to be clearly defined. Both AF and dementia, especially Alzheimer's disease, share multiple risk factors, including old age, diabetes, chronic kidney disease, sleep apnea, hypertension, heart failure, excessive alcohol consumption, and coronary heart disease (13). Thus, it is possible that AF may contribute to cognitive decline through accelerating the underlying pathology of Alzheimer's disease, i.e., the progressive accumulation of beta amyloid and neurofibrillary tangles. In a recent study by Nakase et al, AF significantly correlated with higher periventricular white matter lesions, which may further influence the severity of cognitive impairment in AD patients (14). Accordingly, other pathophysiological players, such as cerebral hypoperfusion, systemic inflammation and coagulation dysfunction may contribute to the link between AF and cognitive impairment beyond stroke (Figure 1).

Cerebral hypoperfusion may be caused by irregular atrial activity and variations in stroke volume, leading to reduced cardiac output (15). As a consequence of cerebral hypoperfusion, dysfunction of neural circuits and cognitive symptoms may ensue. Chronic reduction of cardiac output and related brain hypoperfusion are linked to brain atrophy while acute severe left ventricle dysfunction can cause “watershed” infarcts. Global and/or regional brain (e.g., hippocampal, frontal) volume loss are believed to play a role in the association between AF and dementia (14). Corroborating these assumptions, reduced cardiac output in patients with systolic heart failure (regardless of AF) has been linked to an increased risk of dementia (11).

In elderly patients with cognitive impairment, AF predicts dementia, and ventricular rate response plays a critical role in dementia incidence. Independent studies have suggested that higher ventricular rates lead to progressive increase in critical cerebral hemodynamic events (hypoperfusion and hypertensive events) at the distal cerebral circle (downstream from the middle cerebral artery). Their data have also suggested that a rate control strategy aiming for around 60 beats per minute may be beneficial for cerebral perfusion and cognitive outcomes in persistent AF (16, 17). Studies have also shown lower rates of incident dementia among patients treated with catheter ablation for AF (18, 19). In a meta-analysis, AF catheter ablation was related to significantly lower risks of overall dementia (HR 0.62; 95% CI 0.56–0.68; I2 = 42%), Alzheimer's disease (HR 0.78; 95% CI 0.66–0.92; I2 = 0%) and vascular dementia (HR 0.58; 95% CI 0.42–0.80; I2 = 31%) (20).

Altered hemostatic function (e.g., raised D dimer levels) and inflammation seem to contribute towards this association as well (21). Dementia and AF are both associated with pathological vascular remodeling and systemic inflammation. Studies have described increased levels of inflammatory markers like C reactive protein (CRP) and interleukin (IL)-6 in dementia patients (10). Conversely, elevated CRP levels were independently associated with an increased risk of AF. It is hypothesized that AF may lead to a pro-inflammatory state, making patients more vulnerable to cognitive decline and dementia. Pre-existing inflammation can initiate AF by accelerating the electrical and structural remodeling of the atria via pro-inflammatory cytokines and other mediators (22), contributing to the AF substrate, which subsequently generates an inflammatory response that further enhances atrial remodeling and perpetuates the arrhythmia—the so-called “AF begets AF” phenomenon. AF might also lead to calcium overload in atrial myocytes, resulting in cell death, danger-associated molecular pattern (DAMP) release, and subsequent low-grade inflammatory response activation to repair the damage. However, the underlying mechanisms by which AF induces inflammation remain poorly understood (22).

Statins have been investigated for their potential to reduce vascular inflammation and cognitive decline in AF patients. In a meta-analysis, statins reduced the risk of AF by as much as 30% in postoperative AF and secondary prevention of AF in selected groups (23). Regarding other anti-inflammatory strategies, such as cyclo-oxygenase inhibitors, antioxidants *α*-tocopherol (vitamin E) and selegiline (a monoamine oxidase inhibitor), Ginkgo biloba, propentofylline, their potential anti-dementia and anti-AF effects need to be further explored (22). Studies have examined the impact of anticoagulation therapy, specifically vitamin K antagonists (warfarin) and direct oral anticoagulants (DOACs), on the risk of incident dementia in AF patients (24). Most studies suggested that oral anticoagulant therapy decreased the risk of developing dementia, especially DOACs like dabigatran and rivaroxaban. Warfarin use yields conflicting results when assessing the risk reduction of dementia, with some studies indicating it can increase dementia risk while others suggest the opposite. Overall, maintaining an optimal therapeutic range of INR is associated with a decreased risk of dementia. In summary, DOAC use may result in a lower incidence of dementia compared to treatment with warfarin or no anticoagulant (24).

Given the strong evidence linking AF to dementia, routine screening for cognitive impairment in patients with AF, and vice versa, is important for optimizing management and maybe preventing progression of dementia. Early detection and aggressive rhythm control of AF with ablation or antiarrhythmics, along with control of other risk factors are essential in patients with both conditions. Aerobic exercise, mental/cognitive activity, social engagement, and patient education are suggested to lower the risk of further cognitive decline (25).

## Atrial fibrillation and anxiety/mood disorders: a bidirectional relationship

**Figure 2:**
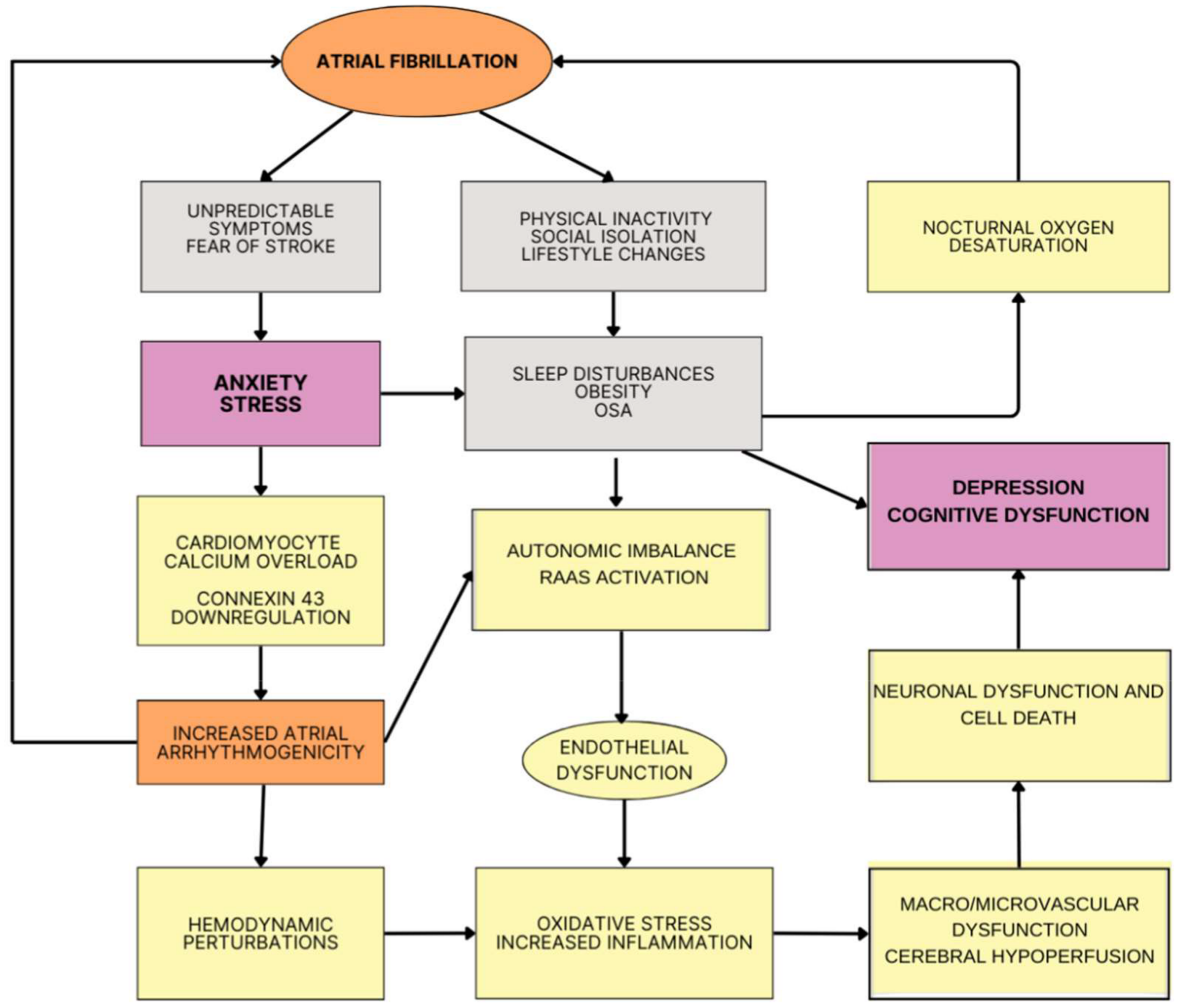
Potential mechanism linking atrial fibrillation (AF) and neuropsychiatric symptoms. Because of the unpredictability nature of AF and the related impact on lifestyle, people with AF can develop stress, anxiety and depression. In parallel to psychosocial mechanisms, increased oxidative stress and inflammation, neurohormonal activation and autonomic dysfunction alongside hemodynamic perturbations (e.g., atrial dilatation, diastolic dysfunction, changes in cardiac output) can lead to hypo-hyper perfusion events and eventually micro/macrovascular changes with cerebral hypoperfusion and, as a consequence, cognitive and behavioral symptoms. OSA, obstructive sleep apnea; RAAS, renin-angiotensin-aldosterone system.

Anxiety and depression are amongst the most frequent psychiatric disorders worldwide, but people with AF are at a higher risk of these psychiatric conditions. This scenario is partly explained by their increased prevalence of overt ischemia (stroke) and covert ischemia (e.g., lacunas, white matter changes) affecting neural circuits involved in emotion processing and regulation (26). Moreover, individuals with AF may experience heightened emotional stress and, as a consequence, anxiety and depression because of the unpredictable nature of AF-related symptoms, such as palpitation and syncope, and constant worry with the risk of stroke and/or bleeding due to anticoagulants (27). (Figure 2) Corroborating these assumptions, the REMEDIAL study was the first clinical randomized trial assessing the impact of AF catheter ablation vs. medical therapy on psychological symptoms. The AF-related burden in the ablation group was lower than in the medical therapy group; this procedure was associated with a very significant improvement in markers of psychological distress, as measured by the Hospital Anxiety and Depression Scale and the Beck Depression Inventory-II, compared with medical therapy at 6 and 12 months follow up (28).

Conversely, anxiety/mood disorders can contribute to AF through different mechanisms. For instance, mood disorders are associated with obesity and sleep problems, including insomnia and sleep apnea with nocturnal oxygen desaturation (29). Hypoxia secondary to intermittent airway obstruction promotes atrial structural remodeling through local and systemic inflammation as well as by mediating oxidative stress. Peripheral chemoreceptor stimulation activates sympathetic reflex activity, predisposing the development of ectopic rhythm and chronic alterations in cardiomyocyte ion channel expression. Negative thoracic pressure events experienced during apnea promote further structural remodeling and ectopic activity through atrial stretch. Baroreceptor stimulation leads to acute atrial effective refractory period shortening, potentially leading to re-entry and atrial arrhythmogenesis (29). Recent studies have also suggested that stress-activated c-Jun N-terminal kinase (JNK), a cardiomyocyte protein kinase that is activated in response to stress, which subsequently phosphorylates the calcium calmodulin kinase (CaMKII) protein, enhancing CaMKII-driven sarcoplasmic reticulum (SRCa2+) calcium mishandling and overload, can trigger a signaling cascade that leads to decreased expression of connexin 43 (Cx43) gap junctions at the genetic level, reducing the number of gap junctions between cardiac cells and predisposing to atrial arrhythmogenesis (30). Of note, S100B, a marker of glial activity, is found to be elevated in serum of patients with major depression and generalized anxiety disorder and also in AF patients undergoing catheter ablation procedures (31). Further research is needed to explore the pathophysiological link possibly mediated by S100B.

There is growing evidence showing that anxiety and depression can increase the risk of AF. In the MESA (Multi-Ethnic Study of Atherosclerosis) study with 6,644 participants who were free of AF at baseline and followed for nearly 13 years, 875 developed AF (13%). Depression at baseline, but not anxiety, chronic stress or anger, was linked to a 35% increased risk for AF (32). In contrast, a diagnosis of PTSD was associated with a 13% increased risk for early incident AF in nearly 1.1 million veterans. People with PTSD usually have unhealthy lifestyle choices and behaviors, such as smoking, drinking, aversion to exercise, unhealthy diet, and drug abuse, which are risks factors for AF. Through persistent stress-related changes in autonomic tone, which can alter atrial electrophysiological characteristics, PTSD may also directly contribute to AF (33).

According to a Holter monitor analysis of nearly 100 people who had either paroxysmal or persistent AF, arrhythmic episodes were more likely to be preceded by negative emotions and less likely by happiness. When compared to other negative emotions, such as rage and anxiety, sadness had the strongest likelihood of preceding an AF episode (32) Nontraditional risk factors, such as psychological features (anger, and hostility) and behavioral/lifestyle factors (e.g., binge drinking, smoking, and vigorous exercise/participation in sports), may have a greater impact on the development of AF in young people than traditional cardiovascular risk factors, which are less prevalent in this age. In young people, 50.2% (average age, 27 years) developed AF without evidence of any underlying cardiovascular comorbidity (33).

Different inflammatory and neurohormonal mechanisms have been implicated in the increased risk of AF related to affective symptoms and disorders (Figure 2). Depression activates the inflammatory response system and enhanced inflammation is associated with incident AF. The effects of depression on the autonomic nervous system (ANS), hypothalamic-pituitary-adrenal (HPA) axis, and renin-angiotensin-aldosterone system can also contribute to increasing the risk of AF. When compared to controls, depressed individuals have higher levels of catecholamines, primarily norepinephrine, indicating increased sympathetic nervous system activity (34). Depression has been linked to hypercortisolism and decreased HPA feedback inhibition (35). Altogether, these depression-related immune/hormonal changes and autonomic dysfunction can make people more vulnerable to AF through changes in the electrophysiologic properties of the atria, such as shortening of atrial action potential duration, increased heart rate variability, abnormal electrical signaling within the cardiac ganglionated plexus and structural remodeling of the atria (36). Anger, anxiety, and stress have also been suggested to elicit AF through similar mechanisms, including direct electrophysiologic changes in the heart and activation of the ANS and HPA axis (37). In a prospective study of over 3,500 Framingham Offspring Study participants, anxiety symptoms were linked to a higher risk of new-onset, postoperative AF in cardiac surgery patients. After adjusting for AF risk factors, tension along with negative emotions of anger and hostility also predicted AF in men (RR = 1.24; 95% CI, 1.04–1.48) (37).

Given that the ANS contributes to AF development and acts as a trigger for AF episodes, modulation of the ANS can be a potential strategy to protect the myocardium from proarrhythmic autonomic effects. It is not simply an increase in parasympathetic tone, but suppression of extreme fluctuations in both components of the ANS, which is believed to be the primary mechanism driving treatment outcomes. The first evidence to support this concept came from a small cohort study evaluating the role of meditation (yoga) in patients with symptomatic paroxysmal AF. Yoga significantly reduced symptomatic and asymptomatic AF burden, improved anxiety and depression, and had a beneficial effect on heart rate and blood pressure (38). Later Stavrakis et al. revealed that low-level cervical vagus nerve stimulation significantly suppressed AF inducibility and shortened AF duration (39). Lampert et al. performed a 12-month electronic diary-based study with 95 patients suffering from paroxysmal AF. Prescription of beta blockers significantly counteracted the AF triggering effects of anger or stress on the sympathetic tone and catecholaminergic surge (40). Nevertheless, antiarrhythmic drugs used in AF have been associated with depression. A meta-analysis including 54 studies with a total number of 212,640 patients with cardiovascular diseases showed that beta blockers were associated with a higher risk of depression (OR: 1.45, 95% CI: 1.26–1.67, *P* < 0.00001) (41).

Antidepressant drugs are known to influence cardiac conduction; specifically tricyclic antidepressants can increase the QT interval (42, 43). In theory, serotonin reuptake inhibition by antidepressants may also predispose to AF through action on 5-HT4 receptors, increase in intracellular calcium and, as consequence, in the amplitude of the pacemaker current in atrial myocytes (44). However, a large UK Clinical Practice Research Datalink did not show increase in the risk of chronic AF with current and/or recent antidepressant use (6–12 months) for depression or anxiety (43). As a word of caution, selective serotonin reuptake inhibitors have been shown to increase the risk of bleeding events in patients with AF who are receiving anticoagulants (45).

## Atrial fibrillation and other neuropsychiatric conditions

AF has been investigated in other neuropsychiatric contexts. Over one million young veterans' electronic health records were examined over a 15-year period showing that AF was more prevalent in people with insomnia (46, 47). Insomnia was also associated with an increased risk of AF in a subsequent study (HR = 1.08, 95% CI: 1.01–1.14) (47). AF was also more likely to occur in men, people over the age of 65, and insomniacs with peripheral artery disease (48). The HPA axis is influenced by sleep disorders, leading to hypercortisolism and risk of AF (35, 47). As mentioned in the previous section, sleep apnea can also cause hypoxia, in addition to increased pulmonary pressure and acidosis, leading to altered cardiac ANS activity, increasing the susceptibility to AF. Conversely, healthy sleep pattern was associated with lower risks of AF (HR comparing extreme categories: 0.71; 95% CI: 0.64–0.80) (47, 48).

In a Taiwanese population-based study, among 927,915 people who had been diagnosed with schizophrenia and bipolar disorder, patients with bipolar disorder had a higher prevalence of AF than patients with schizophrenia (5.5% vs. 3.2%). Patients with BD had more AF possibly due to older age, higher frequency of comorbidities, increased inflammation and serotonin dysfunction. In addition, people with schizophrenia more likely underreported their AF (49). According to the Danish nationwide cohort study, patient with diagnosis of AF and bipolar disorder or schizophrenia are less likely to receive oral anticoagulation therapy, thus have higher risk of complications (50). This finding illustrates the challenges of managing people with comorbid severe mental illnesses and cardiovascular diseases as discussed below (51).

## Rhythm control in neuropsychiatric disorders

A systematic review and meta-analysis including a total of 14 studies and 193,830 AF patients investigated the impact of rhythm control in AF on cognitive function and dementia risk. The group that underwent rhythm control strategies for AF (cardioversion, catheter ablation and/or antiarrhythmic drugs like amiodarone, sotalol, and flecainide) had 38% lower risk of overall dementia as compared to rate control therapy group (using beta-blockers, calcium channel blockers and/or digoxin) (52).

Rhythm control strategies for AF may also be beneficial for individuals experiencing mental health disorders, especially anxiety and depression, as restoring a normal heart rhythm can potentially improve symptoms and quality of life by alleviating the physical discomfort and distress associated with irregular heartbeats (52). Conversely, drugs employed for rhythm control may aggravate psychopathology. For instance, there are case reports of psychosis induced by amiodarone and flecainide. Sotalol can cause depression and other mood changes in up to 10% of individuals (53).

Marked treatment disparities exist in patients with neuropsychiatric disorders and AF. A Finnish retrospective registry-based cohort study showed that the usage of rhythm control strategies in patients with psychiatric disorders, including anxiety, depression and severe mental illnesses (e.g., schizophrenia), was significantly lower that people without any psychiatric disorders (respectively, 17% vs. 23%). The proportion of patients who underwent cardioversion and catheter ablation in the former group was, respectively, 12% and 1.7% as compared to 17.4% and 2.2%, respectively, in the control group (54). Patients with psychiatric disorders are generally managed in primary care settings and less likely referred to definitive treatments for AF because of stigma, economic and other factors (54). Rhythm control treatment strategies like cardioversion, Class III anti-arrhythmic drug therapy and catheter ablation are based on symptom severity, and these patients may face communication and access barriers, influencing physicians' decision to refer to electrophysiologists or specialized centers (54). Other challenges include medication compliance (that can be influenced by polypharmacy and interactions with antidepressants and antipsychotics), limited social or family support (51–54). In this population, addressing the underlying mental health condition is crucial alongside rhythm control strategies.

## Discussion and perspectives

Beyond post-stroke neuropsychiatric complications, there is a growing body of literature indicating a more complex picture related to AF. First, AF is a risk factor for cognitive impairment and dementia independent of stroke. Second, depressive symptoms are strongly associated with AF related burden and are widely under recognized and neglected as a contributor to AF (18, 55). Given that 20% of adults in US study population report depressive symptoms, and that this large population is at increased risk for AF, it will be important to define if identification and treatment of these individuals can reduce the incidence of AF (56). Third, anxiety and stress-related conditions have also been associated with AF. There is considerable data showing that medical interventions (e.g., cognitive therapy and long-term psychotherapy) can be effective in treating these conditions and reducing the associated physiological hyperarousal (57). Whether early detection and successful treatment of stress-related disorders, especially PTSD, can prevent or mitigate the likelihood of developing AF among those exposed to severe trauma also needs to be analyzed in future studies (57). There are unanswered questions regarding the most effective clinical management strategies for patients who suffer from AF and neuropsychiatric conditions. Prospective studies are needed to define the role of anti-depressants in preventing incident AF and improving symptoms and quality of life in AF patients.

As part of the management of AF, the Atrial Fibrillation Better Care Pathway highlighted the need of attention to psychiatric comorbidity (58). To encourage psychopathological assessment during clinical consultations and to provide further empirical evidence of the related impact on AF, recognition by the cardiology community of the role played by psychological factors in the onset and progression of AF is needed. Identifying depression in AF patients, and appropriate therapy may provide therapeutic benefit. Future studies should investigate whether these approaches in newly detected AF individuals can improve AF-related outcomes and quality of life in the short and long-terms. Evidence-based interventions to treat behavioral problems in AF patients are scarce, and non-pharmacological strategies must be investigated. Treatment options aimed at improving coping skills and suppressing interoceptive symptoms can contribute to the balanced modulation of the ANS, which seems to be important for the development of the AF substrate and the onset of AF episodes (5, 55).
